# Guidelines for Conducting Virtual Cognitive Interviews During a Pandemic

**DOI:** 10.2196/25173

**Published:** 2021-03-11

**Authors:** James A Shepperd, Gabrielle Pogge, Jean M Hunleth, Sienna Ruiz, Erika A Waters

**Affiliations:** 1 Department of Psychology University of Florida Gainesville, FL United States; 2 Department of Surgery Division of Public Health Sciences Washington University St. Louis, MO United States

**Keywords:** cognitive interview, COVID-19, guidelines, teleresearch, pandemic, tablet computer, telehealth, training

## Abstract

The COVID-19 pandemic has challenged researchers working in physical contact with research participants. Cognitive interviews examine whether study components (most often questionnaire items) are worded or structured in a manner that allows study participants to interpret the items in a way intended by the researcher. We developed guidelines to conduct cognitive interviews virtually to accommodate interviewees who have limited access to the internet. The guidelines describe the essential communication and safety equipment requirements and outline a procedure for collecting responses while maintaining the safety of the participants and researchers. Furthermore, the guidelines provide suggestions regarding training of participants to use the technology, encouraging them to respond aloud (a potential challenge given that the researcher is not physically present with the participant), and testing and deploying the equipment prior to the interview. Finally, the guidelines emphasize the need to adapt the interview to the circumstances and anticipate potential problems that might arise.

## Introduction

Infectious disease pandemics can potentially derail studies involving in-person interactions with participants, such as cognitive interviews. Cognitive interviews examine whether study components—most often questionnaire items—are worded or structured in a manner that allows study participants to interpret the items in a way intended by the researcher [1]. The interviews allow researchers to fix problems before fielding the survey. Cognitive interviews often involve a “think aloud” protocol where researchers ask participants to think aloud as they read items and reason through their responses. Although the think aloud protocol is challenging and may not be feasible for some participants, it provides insights into how participants interpret items. Researchers can pursue potentially concerning responses with additional verbal questions to identify the point of confusion, and they can explore possible alternative items or instruction wording. Cognitive interviews can also help determine whether certain words, concepts, or phrases are understood similarly across participants, whether a potentially sensitive item is offensive to participants, and whether items require adaptation to accommodate individuals with limited literacy or health literacy.

COVID-19 was declared a national emergency in the United States in March 2020, prompting various social distancing protocols and other restrictions on in-person contact, including a moratorium on in-person human subject research imposed by institutional review boards (IRBs). Videoconferencing platforms including Zoom (Zoom Video Communications), GoToMeeting (LogMeIn Inc), Webex (Cisco Webex), and Skype (Skype Technologies, Microsoft Corp) offer temporary solutions to some researchers in that they facilitate web-based interactions with study participants. However, these platforms require that participants have a web-accessible device, reliable and sufficiently high-speed internet access to support videoconferencing platforms, and a registered account on such platforms and software to access these platforms. Participants with low income and participants who reside at remote locations often do not meet these requirements [2].

At our institutions, COVID-19 led to temporary suspension of all research activities involving human subjects just as we began in-person cognitive interviews for two nationwide surveys. Our participants were caregivers of children with asthma, and many of the caregivers had asthma themselves or other health problems that made in-person interviews hazardous for them. Some of our study staff also had health conditions that placed them at an increased risk of COVID-19–related mortality. In accordance with the IRB restrictions, and to ensure the safety of the participants, their families, and our staff and their families, we developed a minimal contact protocol. The protocol entailed conducting a virtual interview, which can have more advantages than in-person interviews: virtual interviews can be more convenient for researchers and participants; lead to the inclusion of participants who might otherwise be excluded, such as people with disabilities or people who live in remote areas [3]; yield data similar in quality to data obtained through in-person interviews [4]; and—because of the perceived anonymity—facilitate discussions on sensitive topics [5].

We initially developed a protocol to conduct cognitive interviews with caregivers. However, we have since expanded the protocol to interview children with asthma. The challenges researchers encounter undoubtedly vary among studies. We describe some general guidelines that we developed to facilitate successful remote interviews.

## Overview of the Protocol

The cognitive interviews proceeded as follows. Our scheduler called potential participants, invited them to participate in the interview, and then scheduled an interview. The researcher then called the participants the day before the interview to provide them with more details regarding the procedures and safety protocol, and to establish a rapport. The researcher arrived at the participant’s residence and, while still seated in the car, set up the equipment (a laptop, tablet computer, and a portable hotspot device), launched the meeting, and ensured that the tablet displayed the survey and that the tablet screen was visible on all devices involved in the meeting. A research assistant joined the meeting virtually during the setup. Next, the researcher delivered the tablet and portable hotspot device to the participant and returned to the car (or another socially distanced location) to conduct the interview. After the interview, the researcher retrieved the equipment and concluded the meeting. Our sample comprised 8 caregivers (7 women, 1 man; 5 Black individuals, 3 White individuals; aged 33-49 years) of children with asthma and living in Gainesville, FL. All caregivers had low income, but none had impairments that affected their ability to use the equipment. All caregivers had participated in a prior at-home interview with the members of our research group (albeit not the current researcher). None of them declined to participate.

## The Guidelines

### Equipment

We needed two types of equipment: web-enabled communication equipment and safety equipment to prevent the spread of COVID-19. Specifically, we required the following:

1. A survey platform: we used Qualtrics XM (Qualtrics), although SurveyMonkey (SVMK Inc) is equally suitable and free of charge.
2. A portable hotspot device that provides internet access to wireless devices: we purchased a Verizon Jetpack MiFi8800L device (Verizon Wireless) with a monthly contract of US $35.
3. A videoconferencing platform: we used Zoom.
4. A laptop to launch the conference call and to communicate with participants during the interview.
5. Headphones for the laptop to reduce background noise: we used a microphone headset that was previously obtained with an iPhone (Apple Inc) but costs US $11 when purchased separately.
6. A tablet computer for participants to access the internet: we purchased the Apple iPad Air 3rd generation (64 GB, 10.5 inch, Wi-Fi) for US $479 and added a screen protector for US $11, a replacement warranty for US $59, and a protective case (Seymac Co Ltd) for US $19. The screen protector, warranty, and protective case were essential because we could not risk losing the tablet because of accidental damage.
7. Safety equipment: we purchased protective masks, disinfectant wipes, and hand sanitizers, and we placed pens, payment forms, and payment cards (ie, debit cards) in a zip-lock bag.

The total communication equipment cost was US $603 and the total safety equipment cost was US $15.

### Participant Preparation

Most participants had limited or no experience with using the portable hotspot device, tablet computer, web-based videoconferencing platform, or with completing a web-based survey. To ensure a smooth flow of the interview, we called participants the day before the interview and briefed them on the procedures and the safety protocol. We informed them of the number and types of items, noted when the session would begin, how long it would take, how we would compensate them for their participation, and how the tablet computer, hotspot device, survey platform, and videoconferencing platform operated. We also explained our COVID-19 safety protocol: we would wear masks and maintain a distance of 6 feet during interpersonal interaction, and we would use disinfectant wipes to clean the iPad and portable hotspot device before delivering them to and after retrieving them from participants. We acknowledged that although wearing masks and maintaining social distance might feel awkward and uncomfortable, the university required that we adhere to these steps to prevent COVID-19 transmission.

This advance phone call provided us an opportunity to build a rapport with the participants. The researcher underscored the value of the participants’ contribution and emphasized our desire to learn from their expertise as caregivers and our need to receive feedback on how to best ask our survey questions. The researcher endeavored to bond with participants by establishing a friendly tone, a sense of comradery, a shared goal of addressing a health concern, and an understanding that the participants’ views were critical and made a difference (the researcher offered examples of items that were changed based on participant feedback).

### Equipment Preparation

The researcher called participants 1 hour prior to the interview to remind them about the interview, and then arrived at the participants’ residence at least 10-15 minutes early to ensure adequate time to set up the devices and the videoconferencing platform, and to log into the meeting on the laptop and tablet computer. Setting up the equipment required several steps:

1. Turn on the portable hotspot device, tablet computer, and laptop, and ensure that the laptop and tablet computer both access the internet through the portable hotspot device and not from some other wireless device.
2. Open the survey link on the tablet computer. Weblinks to the survey and the web-based meeting can be lengthy. It is often easy to email the links to the tablet computer and then click the links obtained from the email. However, one must take care to log out of the email account before delivering the tablet to participants to prevent participants from accessing the email account through the device. Furthermore, the researcher should also disable any alerts on the tablet computer so that participants are not interrupted during the interview. Instructions for disabling or hiding email accounts on tablet computers are available online.
3. Launch the meeting on the laptop (which allows the researcher to control screen sharing during the meeting) and join the meeting from the tablet computer. Only the researcher and research assistant had access to the URL for the Zoom meeting, which was typically generated 1 hour before the meeting. Although we did not password-protect the meetings, researchers concerned with privacy invasion can do so. In addition, the network connection provided by the portable hotspot device was password protected. Finally, we collected no participant-identifiable information in the survey; Qualtrics encrypts responses using secure socket layers and masks all IP addresses, thus providing the researchers access to only the survey responses.
4. Allow screen sharing from the laptop, and then share the screen for the survey link from the tablet computer. The screen sharing allows the researchers to monitor participants’ responses to the survey in real time and probe them as necessary.
5. Test the audio in the meeting. An audio test can be challenging because of the possibility of generating a feedback loop when the laptop and tablet computer are both logged into the virtual meeting and are proximal to each other. If the sound works properly for both the tablet and the laptop, the researcher can deliver the tablet computer and portable hotspot device to the participant.

The researcher performs these tasks in the car, which requires some juggling. We found it useful to practice setting up the devices in the car at home before proceeding to the participants' residence. The research assistant took notes and asked participants additional questions if needed. Because seeing the researchers’ faces while taking the survey could be distracting and may affect the participant responses, both researchers disabled their cameras throughout the meeting.

### Adapting the Interview to the Circumstances

Once the researcher completed the steps successfully, the researcher delivered the tablet computer and hotspot device to the participant. The researcher did not enter the participant’s residence, but rather stayed outside and cleaned the devices with a disinfectant wipe in front of the participant. If possible, the researcher placed the devices on a porch table or another surface and stepped back rather than handing them directly to the participant. The researcher then introduced the research assistant (who was audible through the tablet) to the participant and answered any questions. This point in time was an opportunity to review the safety protocol with the participant.

After explaining the safety protocol and responding to the participant’s questions, the researcher moved to a distant site (often returning to the car) to proceed with the interview. During this brief transition, the research assistant, speaking through the tablet, reminded the participant of the procedure and reiterated the value of their participation. Many apartment complexes where we conducted the interviews had picnic tables where the researcher could conduct the interviews while the participant completed the survey in their residence. However, sitting outside underscored the need for a microphone headset. In several instances, the researcher was interrupted by other people (eg, the apartment manager, other residents who were being social) while sitting at an outdoor picnic table. Moreover, some apartment complexes were noisy with barking dogs, neighbors talking, and street sounds. Without the microphone headset, these interruptions and noises would be distracting to the researcher and to the participant who can hear through the tablet computer what the researcher hears.

We had only the survey displayed on the tablet computer screen so that we could monitor participants’ responses. We asked participants to read each item on the survey aloud, verbally declare their response, and explain aloud the reason for their response. Reminders were sometimes necessary, yet participants acclimated rapidly to this request even though the researcher was not physically present with them. Each page typically contained 2-10 items, and we stopped participants at the end of each page to probe their responses on the page in more detail and to ask what certain phrases or words meant to them. Having participants talk aloud shortened the interview durations because in many instances, participants had already explained their responses, eliminating our need for further probing.

Once the survey was complete, the researcher retrieved the tablet computer and hotspot device and provided the participant with a zip-lock bag containing a debit card, pen, and payment receipt form. The participant used the pen to sign the payment form, returned the pen and form in the bag, and retained the debit card. As a final gesture, and because the participant handled the zip-lock bag, the researcher offered a squirt of hand sanitizer before leaving. The researcher then returned to the car, sanitized the equipment and their hands, turned off the hotspot device, closed the browsers for the survey and the meeting on the tablet computer, and ended the meeting on the laptop.

### Managing Potential Problems

Unplanned events are inevitable, and the researcher must be prepared to troubleshoot. On two occasions, the portable hotspot device failed: on one occasion, an electrical storm disrupted service for a few seconds, and on another occasion, the portable hotspot device overheated from sustained exposure to the hot sun. In the latter instance, it took a couple of minutes before the hotspot device cooled down and resumed functioning. Occasionally, participants were not wearing masks or wearing them around their chin or neck and the interviewer reminded them to wear a mask or to wear it correctly. We encountered no resistance regarding the safety protocol, perhaps because we were clear in the phone conversations that the university required us to follow the safety protocol, that it was for all our benefit, and that we were all obliged to follow it. In all instances, it was clear that the participant merely forgot to follow the safety protocol.

Portable hotspot devices have limited broadband capacity, and videoconferencing draws considerable bandwidth. If the researcher and participant are both accessing the internet through the hotspot device, they are more likely to experience disrupted internet access. However, these disruptions did not occur for us if only the participant used the video mode and if the researcher closed all other web-based programs on the laptop (such as email platforms). It is noteworthy that Wi-Fi speed is generally low for everyone if too many people in a location attempt to use it simultaneously. Finally, participants sometimes clicked on a button on the tablet computer, which directed them away from the survey, or the tablet computer entered sleep mode during extended periods of conversation (although the audio was still retained throughout the meeting). We addressed all problems rapidly by instructing the participant how to return to the survey. If the researcher had to briefly retrieve the tablet computer or hotspot device, he/she had extra disinfectant wipes for cleaning the surfaces.

We considered having the participant’s face displayed on the screen while they completed the interview, thereby allowing us to monitor their nonverbal responses. Attending to a participant’s nonverbal responses is a vital component of cognitive interviews. Nonverbal cues can reveal confusion (eg, a furrowed brow) and boredom or discomfort (eg, exaggerated sighing). However, we found it challenging enough to monitor their responses to the items, and the video quality was insufficient for monitoring and interpreting facial expressions. Thus, we instead attended closely to participants’ questionnaire responses and their verbalizations. Modulations and inflections in participants’ voices revealed a wealth of useful information (eg, surprise, confusion, incredulity, or annoyance) independent of the content. In addition, participants occasionally took more time to respond to certain survey items, which suggested that they were perhaps struggling with those items. These instances prompted us to ask participants to share with us what slowed them down.

Finally, we were concerned that the participants might be less responsive to researchers conversing with them in an unfamiliar format. However, we encountered no such problems, presumably because of the efforts we took to establish a rapport with participants and because other members of our team had interviewed these participants in the past and thus had established a relationship. We also speculate that participating from one’s own home was comforting, and conversing with remote researchers who could be heard but not be seen generated a sense of privacy and intimacy that fostered greater disclosure.

## Summary

The COVID-19 pandemic has posed challenges among researchers conducting cognitive interviews, particularly in populations with limited access to the internet, an internet accessible device, or web-based videoconferencing platforms. Our guidelines describe how researchers can address these challenges and continue performing studies involving cognitive interviews. These guidelines describe some necessary communication and safety equipment and outline a procedure for collecting responses while maintaining the safety of the participant and researcher. These guidelines also provide tips for establishing rapport, training participants in the technology, encouraging participants to respond aloud, and testing and deploying the equipment prior to an interview. Finally, the guidelines emphasize the need to adapt the interview to various circumstances and anticipate potential unplanned events.

## Abbreviations

IRB: institutional review board
